# Association of Healthy Lifestyles with Non-Alcoholic Fatty Liver Disease: A Prospective Cohort Study in Chinese Government Employees

**DOI:** 10.3390/nu15030604

**Published:** 2023-01-24

**Authors:** Zhen Ling, Chengcheng Zhang, Jun He, Feiyun Ouyang, Dan Qiu, Ling Li, Yilu Li, Xuping Li, Yanying Duan, Dan Luo, Shuiyuan Xiao, Minxue Shen

**Affiliations:** 1 Department of Social Medicine and Health Management, Xiangya School of Public Health, Central South University, Changsha 410008, China; 2 Department of Occupational and Environmental Health, Xiangya School of Public Health, Central South University, Changsha 410078, China; 3 Department of Dermatology, Xiangya Hospital, Central South University, Changsha 430013, China; 4 Furong Laboratory, Changsha 410008, China

**Keywords:** lifestyle factors, combined impact, non-alcoholic fatty liver disease (NAFLD), government employees, cohort

## Abstract

Background: Evidence indicates that certain healthy lifestyle factors are associated with non-alcoholic fatty liver disease (NAFLD). However, little is known about the effect of combined healthy lifestyle factors. Objective: To assess the association of combined healthy lifestyle factors with the incidence of NAFLD. Methods: This cohort study was conducted in Changsha, Hunan Province, China. The healthy lifestyles factors studied were not being a current smoker, having a healthy diet, engaging in physical activity, having a normal body mass index (BMI) and engaging in non-sedentary behavior. NAFLD was diagnosed based on abdominal ultrasonography. Logistic regression models were conducted to investigate the associations being studied. Results: Of the 5411 participants, 1280 participants had NAFLD, with a prevalence of 23.7% at baseline. The incidence of NAFLD among participants without NAFLD at baseline was found to be 7.2% over a mean follow-up of 1.1 years. Compared with participants with 0–1 low-risk factors, the OR of NAFLD was 0.50 (95% CI: 0.29–0.82, *p* = 0.008) for those with at least 4 low-risk factors. Similar associations were observed in subgroup analyses and sensitivity analyses. Conclusion: This study suggests that a combined healthy lifestyle pattern may considerably decrease the risk of NAFLD in Chinese government employees.

## 1. Introduction

Non-alcoholic fatty liver disease (NAFLD) is one of the most common chronic liver diseases in the world, affecting approximately 25% of the general population. NAFLD has expanded into a global public health issue and imposes a substantial economic burden on all societies [1]. Increasing evidence indicates that NAFLD may develop into end-stage liver disease [2] (e.g., decompensated cirrhosis, severe hepatitis, advanced liver cancer, etc.). In addition, it can contribute to an increased risk of multisystem disease, including cardiovascular events, metabolic disorders and kidney problems, by affecting extra-hepatic organs and regulatory pathways [3]. Previous research has observed that the prevalence of NAFLD parallels urbanization and industrialization trends, and lifestyle changes have been found to be associated with the NAFLD epidemic worldwide, especially in the Asia-Pacific region [4].

Having an unhealthy lifestyle, a common and crucial modifiable risk factor, has been proven to be independently related to many chronic noncommunicable diseases, such as type 2 diabetes [5], cancer [6] and cardiovascular disease [7]. In addition, a recent study suggested that maintaining a healthy lifestyle may contribute to a higher life expectancy [8]. Meanwhile, many studies have shown that smoking [9], physical inactivity [10], sedentary behavior [11,12], higher weight [13] and an unhealthy diet [14] were individually related to an increased risk of NAFLD. For example, replacing saturated fatty acids with plant-based polyunsaturated fatty acids in the diet reduces serum insulin, the total/high-density lipoprotein cholesterol ratio, low-density lipoprotein cholesterol, triglycerides and liver fat [15]. However, most of these studies only investigated the relationship between individual healthy lifestyle factors with NAFLD, thus ignoring the fact that many lifestyle factors often coexist. It is necessary, therefore, to evaluate the joint effects of these modifiable healthy lifestyle factors on the risk of NAFLD. Further, less is known about the possible benefit of combined healthy lifestyle factors with regards to the risk of NAFLD, particularly in developing countries such as China.

Given this, we combined five modifiable healthy lifestyle factors recommended in previous studies, namely, smoking, physical activity, BMI, sedentary behavior and diet, in order to further assess the relationship between combined healthy lifestyle factors and NAFLD. We set out to clarify the following aims in this study: to investigate the relationship between a combination of healthy lifestyle factors and the risk of NAFLD, and how this relationship differs across subgroups.

## 2. Methods

### 2.1. Research Design and Study Participants

**Figure 1:**
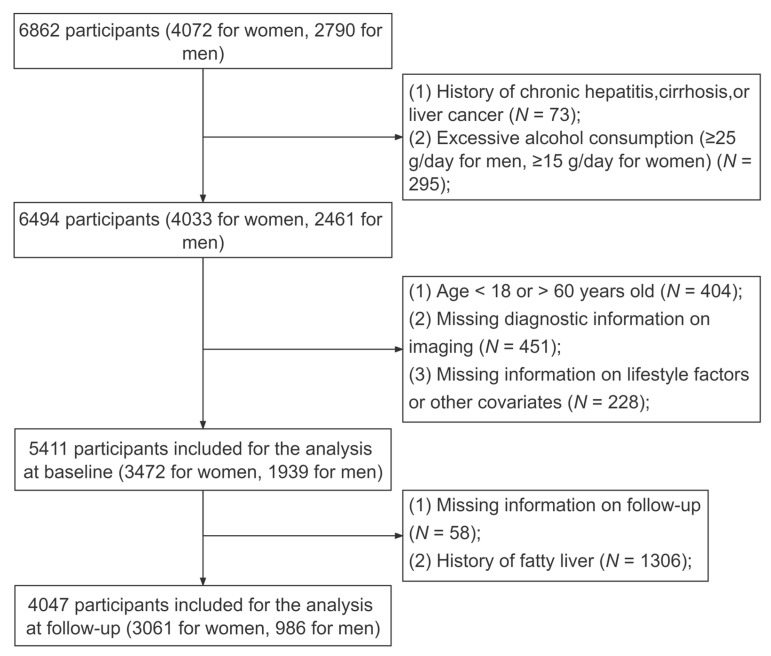
Flowchart of the population included in our analyses.

The research involved a prospective cohort study conducted in Changsha City, Hunan Province, which aimed to investigate chronic diseases in Chinese government employees. Government employees in China are those who carry out legal public duties in the national legislative branch, judicial branch, administrative organs, Communist Party of China Party organs, democratic parties, people’s organizations and public institutions. In our research, participants were mainly from government departments, universities and hospitals with a predominantly sedentary work style. The baseline survey consecutively enrolled a total of 6862 participants from 10 government organizations through stratified cluster sampling from January 2018 to April 2020. In addition, we conducted a 1-year follow-up survey for government employees who participated in the baseline survey [16]. All employees filled out an online questionnaire via a cellphone or tablet prior to the ultrasound examination. Detailed information and data collection procedures have been published in previous studies [17,18]. We excluded participants who had a previous history of chronic hepatitis, cirrhosis, liver cancer (*N* = 73) or excessive alcohol consumption (Males: ≥25 g per day; females: ≥15 g per day) (*N* = 295). In addition, participants with missing ultrasound diagnosis data (*N* = 451) and other related covariate data (*N* = 228), as well as those below 18 or above 60 years of age (*N* = 404) at baseline, were excluded. As a result, 5411 participants (3472 women, 1939 men) were included in the analyses at baseline. However, 58 employees refused to participate in the follow-up survey. After excluding those with a history of fatty liver (*N* = 1306) at baseline, 4047 employees (3061 women, 986 men) were included in the final analyses (Figure 1).

### 2.2. Assessment of Healthy Lifestyle Factors

A structured online questionnaire evaluated a range of healthy lifestyle factors at baseline. For cigarette smoking, it asked all respondents the following question: Do you smoke, at least one cigarette per day for more than a half-year? (there were 3 response categories: never, former or current). Further, we collected information on current smokers regarding the amount of cigarettes they smoked per day and the age at which they started smoking. For alcohol consumption, the questionnaire asked all employees the following question: Do you consume alcohol, at least once per week for more than a half-year? (there were 3 response categories: never, former or current). Information about alcohol consumption included the frequency current drinkers drank per week, the volume of alcohol they consumed per drinking session and the types of alcohol they consumed. Regarding a healthy diet, the questionnaire asked: How frequently have you eaten the following foods in the last 12 months? (Eating is considered to have occurred once in one meal; select the corresponding frequency). The details are shown in Table S1 in Supplementary Materials. Information on sedentary behavior [19] involved the time participants spent sitting or reclining in activities (e.g., screen-time, reading, playing cards or mahjong, etc.) engaged in during their leisure time a day [20]. For physical activity, the questionnaire asked the following question: How frequently and for how long have you engaged in physical activities in the last 12 months? The usual types and durations of activities were also collected. The participants’ daily physical activity levels were calculated based on previous literature [21]. All participants’ heights, weights and waist circumferences were assessed by skilled researchers using industry-recognized tools. The following formula was used to determine BMI: BMI = weight/height^2^ (kg/m^2^).

### 2.3. Healthy Lifestyle Definition

Five healthy lifestyle factors were identified and combined in order to form our healthy lifestyle definitions, namely, smoking, diet quality, sedentary behavior, physical activity and BMI according to the American Heart Association (AHA) and previous studies [9,11,14,22].

For smoking, we defined non-smoker participants and those participants who had not smoked for more than half a year as belonging to the healthy group. For diet intake, according to the recommendations of Dietary Guidelines for Chinese Residents (2022) and the actual situation of this cohort, we considered 5 types of food items, namely, red meat, vegetables, fruits, soybean and products, and fish. A participant’s diet was defined as healthy if it included more than 4 of the healthy diet food items. Information on the construction of the healthy diet score is displayed in Supplementary Materials Table S2. Non-sedentary behavior was defined as ≤2 h per day of sitting during leisure time [23]. For physical activity, we assigned participants to the healthy group if they were higher than or equal to a sex-specific upper quarter with regards to their physical activity level. Finally, we used two methods to define a healthy weight [24]. The first involved assigning participants with a BMI of 18.5 to 23.9 kg/m^2^ to be in the healthy group. The other was assigning participants to the healthy group if their waist measurements were less than 80 cm for women and 85 cm for men [24]. The details are shown in Supplementary Materials Table S3.

### 2.4. Diagnosis of NAFLD

All employees in our study were examined by a qualified physician using abdominal ultrasonography, and the diagnostic standard was based on the Chinese Association of Liver Diseases recommendations [25]. Diagnosing NAFLD involved the following five criteria: (1) Diffuse enhancement of the near-field echogenicity in the hepatic region that is stronger than that in the kidney and spleen regions, with there being gradual attenuation of the far-field echogenicity; (2) Poor visualization of intrahepatic luminal structures; (3) Mild to moderate hepatomegaly, with rounded and blunted liver margins; (4) Reduced or even difficult to visualize blood flow signal in the liver on color Doppler ultrasound, but normal blood flow distribution; and (5) Poorly visualized or incomplete right lobe of the liver and diaphragm.

### 2.5. Assessment of Covariates

The following variables were considered as covariates: (1) categorical variables: gender (male or female), employment grade (primary, intermediate or senior/deputy senior), educational levels (high school or below, undergraduate or postgraduate or above), marriage status (divorced/widowed, single or married), annual household income (<100,000, 100,000–300,000 or >300,000 CNY), alcohol consumption (current or never/former), and a history of using statins (yes or no); and (2) continuous variables: age, total cholesterol (TC), triglycerides (TG), fasting plasma glucose (FPG), total bilirubin (TBIL), high-density lipoprotein cholesterol (HDL-C) and low-density lipoprotein cholesterol (LDL-C).

Covariate information was estimated through questionnaires, including sociodemographic factors and family medical history information. Blood samples, following a 12 h fast, were taken and transmitted immediately to the medical laboratory for biomarker analysis. FPG levels were measured by the glucose oxidase method, TBIL levels were measured by the diazobenzene sulfonate method and TC, TG, HDL-C and LDL-C levels were measured by the standard enzyme method. All determinations were carried out with a Chemistry System Autoanalyzer (Hitachi 7600-110; Tokyo, Japan) in the Medicine Laboratory Department of the Third Xiangya Hospital, which has been accredited by the Chinese Society of Laboratory Medicine.

### 2.6. Statistical Analyses

The basic characteristics of the qualitative variables of the participants were described using percentages, and means and standard deviations (SDs) were used to express the quantitative variables. Chi-square tests for qualitative variables and analysis of variance (ANOVA) for quantitative variables were used to compare differences in basic characteristics. Contingency coefficients were used to estimate the pairwise correlation of healthy lifestyle factors.

We performed the multivariable logistic regression method to assess the association between a combination of lifestyle factors and the risk of NAFLD. We employed three regression models, namely, a crude model (unadjusted model) and two adjusted models (model 1 and model 2). To assess the association between a combination of healthy lifestyle factors and NAFLD, model 1 did not correct for any covariates, and model 1 adjusted for age, gender, education level, marriage status and employment grade. Model 2, the full model, was employed by additionally adjusting for a history of using statins, as well as TC, TG, FPG, TBIL, HDL-C and LDL-C. Potential confounders that were analyzed were based on the previous literature and our work with the data [26,27].

Further, to estimate the consistency of our findings, sensitivity analyses were performed. As waist circumference is often used as an indicator of central adiposity, we used WC instead of BMI to construct the combined lifestyle factors for comparison. Then, the second sensitivity analysis was carried out, which only included non-current drinkers. In addition, we stratified the analysis by gender, age, marriage status and socioeconomic status using logistic regression.

The primary statistical analyses were carried out in R version 4.1.1. Specifically, the “glmnet” package was used for the logistic regression, and all figures in the article were implemented using the “ggplot2” and “foreign” packages. A two-tailed *p* < 0.05 was used to determine the statistical significance.

## 3. Results

### 3.1. Basic Characteristics of Study Participants

Table 1 provides the basic characteristics of the participants, which are grouped by the combined healthy lifestyle factors. Of the 5411 participants (1939 men and 3472 women, with an average age of 38.19 years), 521 (9.6%), 1390 (25.7%), 2201 (40.7%) and 1299 (24.0%) had at least four and five, three, two, one and zero healthy lifestyle factors, respectively. A total of 1280 participants had NAFLD, with the disease having a prevalence of 23.7% at baseline. Its prevalence decreased as the number of combined healthy lifestyle factors increased, with participants with only 0–1 healthy lifestyle factors having the highest prevalence of NAFLD. The participants who had more healthy lifestyle factors than the other participants were females, with higher levels of education and income, and lower levels of triglycerides, total cholesterol, fasting rapid glucose, low-density lipoprotein cholesterol, platelet count, albumin and alanine aminotransferase, as well as a higher level of high-density lipoprotein cholesterol than other participants. All basic characteristics, except education level and family income, differed markedly between the healthy lifestyle factor groups (*p*-values < 0.05). There were 341 participants who were diagnosed with NAFLD over a period of 4761.5 person-years, and the median follow-up time was 1.1 years. In addition, the prevalence of metabolic syndrome was 47.5% in the NAFLD group, compared to 5.5% in the non-NAFLD group (*N* = 5134). Among the 5411 participants, 4897 (90.5%) were non-current smokers, 1405 (26.0%) chose non-sedentary behavior, 3224 (59.6%) had a normal BMI, 938 (17.3%) maintained a healthy diet and 1376 (25.4%) were active in physical activity. See Table S4 for details. Table S5 provides details about the combination of lifestyle factors and includes 32 combinations in total. The most frequent combinations were the not currently smoking and normal BMI group and the no current smoking group, corresponding to 26.1% and 16.8%, respectively.

### 3.2. Associations between Combined Lifestyle Factors and NAFLD

The associations between single lifestyle factors and the risk of NAFLD are displayed in Table S6. The univariate logistic analysis found that, among the five individual risk factors, BMI, leisure-time sedentary behavior and physical activity were most associated with NAFLD, with these having corresponding ORs of 0.12 (95% CI: 0.10–0.14, *p* < 0.001), 0.78 (95% CI: 0.66–0.91, *p* = 0.002) and 0.84 (95% CI: 0.71–1.00, *p* = 0.045), respectively.

**Figure 2:**
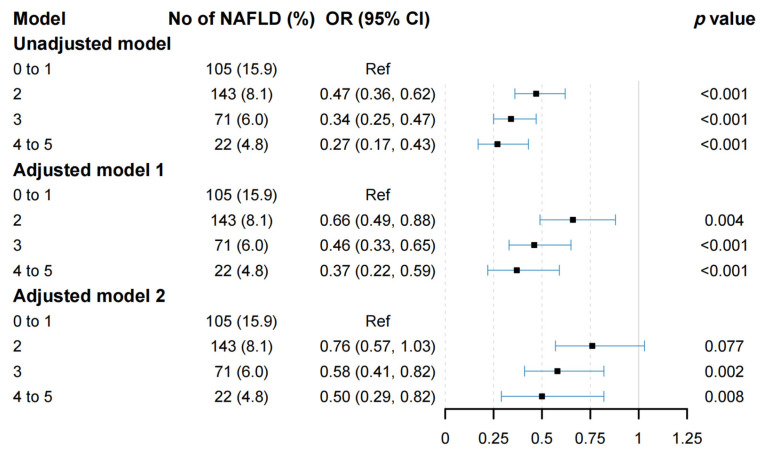
Logistic regression of healthy lifestyle scores and NAFLD. NAFLD, non-alcoholic fatty liver disease; OR, odds ratio; CI, confidence interval.

A multivariable logistic regression model was carried out to examine the relationship between the combined healthy lifestyle factors and the risk of NAFLD. As indicated in Figure 2, compared to the group living unfavorable lifestyles (0 to 1 healthy lifestyle factors), the risk of developing NAFLD was reduced by 53% (OR = 0.47, 95% CI: 0.36–0.62, *p* < 0.001), 66% (OR = 0.34, 95% CI: 0.25–0.47, *p* < 0.001) and 73% (OR = 0.27, 95% CI: 0.17–0.43, *p* < 0.001) in the groups with two, three and at least four healthy lifestyle factors, respectively. Model 1 showed the same trends after adjusting for age, gender, education level, marital status and employment grade, with the corresponding ORs of 0.66 (95% CI: 0.49–0.88, *p* = 0.004), 0.46 (95% CI: 0.33–0.65, *p* < 0.001) and 0.37 (95% CI: 0.22–0.59, *p* < 0.001) obtained, respectively. Based on model 1, additional adjustment for a history of using statins, as well as TC, TG, FPG, TBIL, HDL-C and LDL-C, did not substantially change the associations found (OR for two healthy lifestyle factors: 0.76, 95% CI: 0.57–1.03, *p* = 0.077; OR for three healthy lifestyle factors: 0.58, 95% CI: 0.41–0.82, *p* = 0.002; OR for at least four healthy lifestyle factors: 0.50, 95% CI: 0.29–0.82, *p* = 0.008).

### 3.3. Analyses of Subgroups and Sensitivity

**Figure 3:**
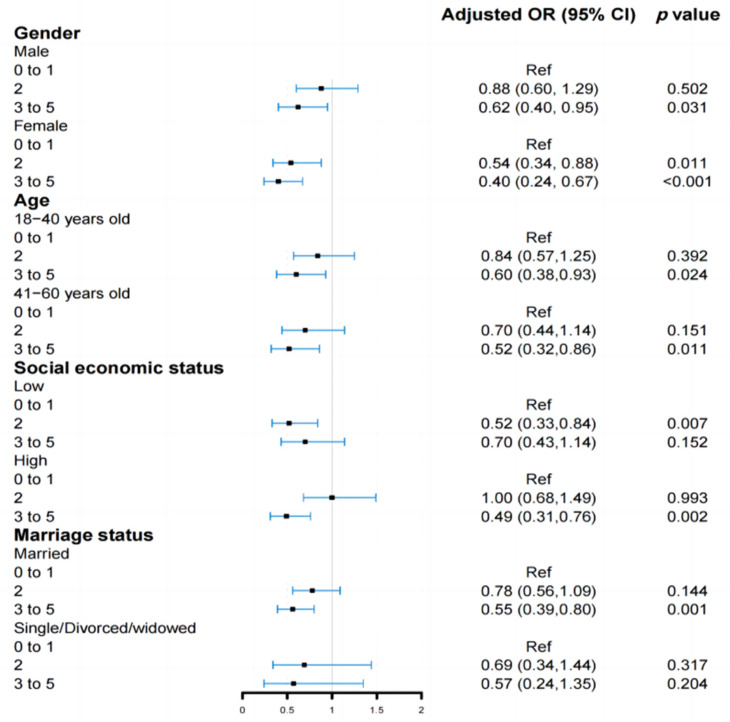
Stratification analyses of the association between healthy lifestyle scores and NAFLD. OR, odds ratio; CI, confidence interval.

Figure 3 displays the outcomes of the subgroup analyses. Compared with one or below factors, a significantly monotonical association between healthy lifestyles (≥3 factors) and NAFLD was observed among the subgroups, with an OR of 0.62 (95% CI: 0.40–0.95, *p* = 0.031) obtained for males, 0.40 (95% CI: 0.24–0.67, *p* < 0.001) for females, 0.60 (95% CI: 0.38–0.93, *p* = 0.024) for participants aged 18 to 40 and 0.52 (95% CI: 0.32–0.86, *p* = 0.011) for participants aged 41 to 60. A significantly inverse association between healthy lifestyles (≥3 factors) and NAFLD was also maintained in the high socioeconomic status and married subgroups, with the adjusted OR values and 95% confidence intervals being 0.49 (95% CI: 0.31–0.76, *p* = 0.002) and 0.55 (95% CI: 0.39–0.80, *p* = 0.001), respectively. When the healthy lifestyles were constructed using waist circumference rather than BMI, the inverse relationship between the healthy lifestyle factors and the risk of NAFLD was not substantially altered. In the final model, compared to the group having unfavorable lifestyles (0 to 1 healthy lifestyle factor), the risk of NAFLD was reduced by 52% (OR= 0.48; 95% CI: 0.35–0.65, *p* < 0.001), 52% (OR = 0.48, 95% CI: 0.34–0.68, *p* < 0.001) and 48% (OR = 0.52, 95% CI: 0.32–0.82, *p* = 0.005) in the groups with two, three and at least four healthy lifestyle factors, respectively (Table S7). We also noticed a significantly monotonical relationship between the increased healthy lifestyle factors and decreased risk of NAFLD among non-current drinkers, with an adjusted OR of 0.72 (95% CI: 0.53–0.99, *p* = 0.044) obtained for two healthy lifestyle factors, 0.54 (95% CI: 0.38–0.79, *p* = 0.001) for three healthy lifestyle factors and 0.51 (95% CI: 0.30–0.84, *p* = 0.010) for at least four healthy lifestyle factors, respectively (Table S8). Furthermore, Table S9 shows the weak correlations (r < 0.15) found between elements of the combined healthy lifestyle factors.

## 4. Discussion

In the present prospective follow-up study, we discovered a strong association between participants having a combined healthy lifestyle, which included their not currently smoking, having a healthy diet, engaging in regular physical activity, having a normal BMI and engaging in non-sedentary behavior, and a significantly lower risk of NAFLD. Participants who had two or more healthy lifestyle factors exhibited a reduction in their relative risk of NAFLD, ranging from 24% to 73%, compared to those having unhealthy lifestyles (0 to 1 healthy lifestyle factor).

Previous observational studies have demonstrated that individual lifestyle factors are of critical importance in the progression of NAFLD [9,10,11,12,28,29]. To the best of our knowledge, however, only a relatively small number of studies have looked into the relationship between combinations of lifestyle factors and NAFLD. A community-based cross-sectional study found that middle-aged and older Chinese persons with better healthy lifestyle scores—including nonsmoking, moderate BMI, periodic exercise and healthy food intake—had reduced prevalence of NAFLD, recording a corresponding OR value of 0.35 (95% CI: 0.25–0.51) [26]. A 22.83-year follow-up study in the National Health and Nutrition Examination Survey (NHANES) III showed that adopting a healthy lifestyle, which was defined as eating well, exercising regularly, quitting smoking and abstaining from alcohol, resulted in a 36% lower all-cause mortality among NAFLD patients, in comparison to the unfavorable lifestyle group [30]. Another study from NHANES found a significant association between five favorable lifestyle items, namely, dietary patterns, BMI, physical activity, smoking and sleep status, and NAFLD and fibrosis in a representative sample of US adults [31]. Further proof that lifestyle changes can greatly reduce the severity of steatosis and liver impairment has been revealed by clinical trials in NAFLD populations in China, Europe and the USA [32,33,34,35]. Similar to the previous studies, the present study demonstrates that adopting a healthy lifestyle may lower NAFLD incidence among the general public, a measure which has great potential in the primary prevention of NAFLD.

For both males and females, adopting a healthy lifestyle may greatly lower the risk of NAFLD. Gender roles and social norms lead to different lifestyle risk factors for men and women, such as unhealthy lifestyles like smoking, alcohol consumption and other risk-taking behaviors, which are widely regarded as desirable male norms in most parts of the world [36]. Evidence from prior epidemiological research also strongly suggests that males and females differ in terms of their risk factors for NAFLD. Among general adult populations, males have been found to be more likely to have NAFLD than females, mostly because they were more likely to be obese, have diabetes and metabolic syndrome, and engage in poor lifestyle behaviors, such as smoking, eating poorly and not exercising [37,38,39,40]. When further considering specific age groups, the incidence of hepatic steatosis has been found to be similar in postmenopausal women compared to men [41]. Similar to this previous research, our findings from the current study support the notion that women have a lower relative risk of developing NAFLD than men.

In this study, we found that obesity was the major contributor to the risk of NAFLD, which is consistent with the findings of earlier studies [42,43,44]. Waist circumference measurement may be a more reliable method of examining visceral obesity’s relationship with the likelihood of developing NAFLD, given its significance as a key risk factor for metabolic syndrome complications [45,46]. Interestingly, we found that, when we constructed the healthy lifestyle using WC rather than BMI, the most favorable lifestyle group had a lowered NAFLD risk of 48% compared to the unfavorable group (the original reduction was 50%). In addition, weight reduction has been found to be extremely beneficial in reducing liver steatosis and fibrosis, even in those who already had NAFLD [14,47,48]. Maintaining physical activity, watching one’s diet and reducing sedentary behavior are all essential for weight loss and maintenance. We found that physical exercise and sedentary behavior were both independently associated with the likelihood of developing NAFLD, which is also supported by data from earlier studies [11].

The study we conducted has a number of strengths. NAFLD, the outcome variable of interest, was diagnosed by specialists via abdominal ultrasonography. The prospective cohort study’s large sample size allowed for the detection of evidence of the relationship between a healthy lifestyle and a lower risk of NAFLD. Further, using a combined healthy lifestyle enabled us to comprehensively characterize an individual’s profile. Despite these advantages, there are several limitations to our research. First, the study had a median follow-up of 1.1 years, which might have been associated with reverse causation; this needs to be explained by further long-time cohort studies. In addition, our study population was not typical of the entire population since it excluded workers from rural areas, the jobless and people with unstable employment. As a result, the generalizability of our findings may be constrained. Third, the questions in the lifestyle factors questionnaire utilized in this investigation were modified and used in numerous other studies, rather than being directly validated. Nonetheless, such misclassification may have led to weakened associations. Additionally, recollection bias was not able to be totally eliminated from our study, and lifestyle characteristics that were only examined once at baseline might not have accurately reflected long-term exposure. Accordingly, these factors might have affected the risk estimates. More studies are required to evaluate the benefits of adopting healthy lifestyles over time with regards to lowering the risk of NAFLD. There may still have been residual confounding factors from additional unmeasured or unidentified factors, even when all the models were corrected for recognized potential biases.

## 5. Conclusions

In conclusion, our findings provide robust evidence that adopting a healthy lifestyle pattern—including quitting smoking, maintaining a normal BMI, eating a balanced diet, cutting back on sedentary behavior and engaging in physical activity—can lower the risk of NAFLD. From a public health viewpoint, lifestyle modifications have great potential in the primary prevention of NAFLD. Further evidence of the effectiveness of comprehensive lifestyle modification in the prevention of NAFLD is expected to come from the continuing follow-up of this prospective cohort study.

## Figures and Tables

**Table 1:** Characteristics of the baseline study.

Characteristics	Total	Healthy Lifestyle Score				*p* Value
		0–1	2	3	4–5	
*N*	5411	1299	2201	1390	521	
Age (years), mean ± SD	38.19 ± 9.32	38.48 ± 9.66	37.03 ± 9.03	38.86 ± 9.21	40.60 ± 9.28	<0.001
Gender, n (%)						<0.001
Female	3472 (64.2)	500 (38.5)	1527 (69.4)	1034 (74.4)	411 (78.9)	
Male	1939 (35.8)	799 (61.5)	674 (30.6)	356 (25.6)	110 (21.1)	
Education level, *n* (%)						0.057
High school or below	252 (4.7)	77 (5.9)	89 (4.0)	69 (5.0)	17 (3.3)	
University	2704 (50.0)	648 (49.9)	1129 (51.3)	673 (48.4)	254 (48.8)	
Postgraduate or above	2455 (45.4)	574 (44.2)	983 (44.7)	648 (46.6)	250 (48.0)	
Marriage status, *n* (%)						<0.001
Divorced/widowed	137 (2.5)	26 (2.0)	50 (2.3)	46 (3.3)	15 (2.9)	
Single	854 (15.8)	222 (17.1)	405 (18.4)	181 (13.0)	46 (8.8)	
Married	4420 (81.7)	1051 (80.9)	1746 (79.3)	1163 (83.7)	460 (88.3)	
Grades of employment						<0.001
Primary	2174 (40.2)	550 (42.3)	953 (43.3)	518 (37.3)	153 (29.4)	
Intermediate	2117 (39.1)	456 (35.1)	852 (38.7)	590 (42.4)	219 (42.0)	
Senior/deputy senior	1120 (20.7)	293 (22.6)	396 (18.0)	282 (20.3)	149 (28.6)	
Annual household income (CNY), *n* (%)						0.127
<100,000	1850 (34.2)	470 (36.2)	755 (34.3)	457 (32.9)	168 (32.2)	
100,000–300,000	3040 (56.2)	703 (54.1)	1253 (56.9)	795 (57.2)	289 (55.5)	
>300,000	521 (9.6)	126 (9.7)	193 (8.8)	138 (9.9)	64 (12.3)	
Alcohol						<0.001
Current	303 (5.6)	146 (11.2)	96 (4.4)	54 (3.9)	7 (1.3)	
Never/former	5108 (94.4)	1153 (88.8)	2105 (95.6)	1336 (96.1)	514 (98.7)	
History of using statins, *n* (%)	39 (0.7)	17 (1.3)	11 (0.5)	7 (0.5)	4 (0.8)	0.033
TC (mmol/L), mean ± SD	4.77 ± 0.91	4.92 ± 0.96	4.73 ± 0.90	4.74 ± 0.88	4.68 ± 0.90	<0.001
TG (mmol/L), mean ± SD	1.35 ± 1.19	1.82 ± 1.62	1.26 ± 1.03	1.14 ± 0.90	1.08 ± 0.81	<0.001
FPG (mmol/L), mean ± SD	5.32 ± 0.88	5.49 ± 1.09	5.26 ± 0.81	5.28 ± 0.83	5.22 ± 0.54	<0.001
TBIL (μmol/L), mean ± SD	13.34 ± 4.91	12.90 ± 4.42	13.31 ± 4.82	13.65 ± 5.26	13.72 ± 5.38	<0.001
HDL-C (mmol/L), mean ± SD	1.44 ± 0.33	1.30 ± 0.31	1.46 ± 0.33	1.51 ± 0.32	1.53 ± 0.32	<0.001
LDL-C (mmol/L), mean ± SD	2.72 ± 0.76	2.79 ± 0.81	2.69 ± 0.75	2.72 ± 0.73	2.66 ± 0.73	0.001
PLT (10^9^/L), mean ± SD	224.95 ± 51.39	227.11 ± 50.78	225.93 ± 51.74	222.37 ± 50.34	222.32 ± 53.88	0.047
ALB (g/L), mean ± SD	45.44 ± 3.03	45.84 ± 3.17	45.36 ± 3.06	45.36 ± 2.88	45.00 ± 2.88	<0.001
ALT (U/L), mean ± SD	21.68 ± 18.85	28.99 ± 27.76	20.11 ± 15.19	18.67 ± 13.36	18.18 ± 11.82	<0.001
Ultrasound-based NAFLD, *n* (%)	1280 (23.7)	618 (47.6)	414 (18.8)	189 (13.6)	59 (11.3)	<0.001

SD, standard deviation; CNY, Chinese yuan; TC, total cholesterol; TG, triglyceride; FPG, fasting plasma glucose; TBIL, total bilirubin; HDL-C, high-density lipoprotein cholesterol; LDL-C, low-density lipoprotein cholesterol; PLT, platelet count; ALB, albumin; ALT, alanine aminotransferase; NAFLD, non-alcoholic fatty liver disease.

## Data Availability

The data used in this study are available on request from the corresponding author.

## Supplementary Materials

The following supporting information can be downloaded at: https://www.mdpi.com/article/10.3390/nu15030604/s1, Table S1: Food frequency questionnaire; Table S2: Diet score extended information; Table S3: Criteria of Healthy Lifestyle Score in the present study; Table S4: The prevalence of the individual risk factors; Table S5: Details about combination of lifestyle factors; Table S6: Association between single risk factors and risk of NAFLD; Table S7: The lifestyle that included waist circumference rather than BMI and risk of NAFLD; Table S8: Association of healthy lifestyle scores and risk of NAFLD among non-current drinkers; Table S9: Contingency coefficient between individual lifestyle factors.
