# Supplementary material for: Association of Healthy Lifestyles with Non-Alcoholic Fatty Liver Disease: A Prospective Cohort Study in Chinese Government Employees
Source: Nutrients. 2023 Jan 24;15(3):604. doi: 10.3390/nu15030604 (PMC9921275; doi:10.3390/nu15030604)
Supplement: Supplementary file 1 [file nutrients-15-00604-s001.zip › nutrients-2117524-supplementary.pdf]

Table S1. Food frequency questionnaire.

**In the past year, how often do you usually eat the following foods? (once in one meal is considered to have eaten; choose the corresponding frequency)**					
Foods	Not at all/rarely	Less than once per week	1-3 times per week	4-6 times per week	Every day
Rice					
Wheat					
Grains					
Fish					
Vegetables					
Poultry meat					
Livestock meat (pork, beef/lamb. etc.)					
Soybean and products					
Fruits					
Eggs					
Dairy products					
Nuts					
Desserts					
Pickle food					
Smoked fish or meat					

Table S2. Diet score extended information.

**Diet Complement**	**Intake Goal**
Fruit	Consumption every day
Vegetable	Consumption every day
(Shell)fish	≥ 2 days per week
Soybean and product	≥ 4 days per week
Processed meats and red meat	< 7 days per week

Healthy diet patterns were adapted from the Dietary Guidelines for Chinese Residents (2022). The healthy diet score was dichotomized as 1 = at least 4 of the healthy food items, and 0 = fewer than 4 of the healthy food items.

Table S3. Criteria of Healthy Lifestyle Score in the present study.

**Healthy Lifestyle Factor**	**Score**
Smoking	
Current smokers	0
Never, occasional or former smokers	1
Dietary quality	
Unhealthy diet quality: 0-3 points for alternate Dietary Guidelines for Chinese Residents (2022) score	0
Healthy diet quality: 4-5 points for alternate Dietary Guidelines for Chinese Residents (2022) score	1
BMI	
BMI < 18.5 or ≥ 24.0 kg/m^2	0
18.5 ≤ BMI < 24.0 kg/m^2	1
Physical activity	
Inactive: lower than a sex-specific upper quarter of the physical activity level	0
Active: higher than or equal to a sex-specific upper quarter of the physical activity level	1
Leisure-time sedentary behavior	
≥ 2 hours/day	0
< 2 hours/day	1

Table S4. The prevalence of the individual risk factors.

Lifestyle Factor Score	Total (%)	Normal (%)	NAFLD (%)	*P* value
N	5411	4131	1280	
Smoking				<0.001
0	514 (9.5)	246 (6.0)	268 (20.9)	
1	4897 (90.5)	3885 (94.0)	1012 (79.1)	
Leisure-time sedentary behavior				0.042
0	4006 (74.0)	3030 (73.3)	976 (76.2)	
1	1405 (26.0)	1101 (26.7)	304 (23.8)	
BMI				<0.001
0	2187 (40.4)	1155 (28.0)	1032 (80.6)	
1	3224 (59.6)	2976 (72.0)	248 (19.4)	
Dietary quality				1.000
0	4473 (82.7)	3415 (82.7)	1058 (82.7)	
1	938 (17.3)	716 (17.3)	222 (17.3)	
Physical activity				0.142
0	4035 (74.6)	3101 (75.1)	934 (73.0)	
1	1376 (25.4)	1030 (24.9)	346 (27.0)	

Table S5. Details about combination of lifestyle factors.

Combination of Lifestyle Factors	Total (%)
No current smoking and normal BMI	1415 (26.1)
No current smoking	913 (16.8)
Normal BMI, non-sedentary behavior and no current smoking	461 (8.5)
Physical activity, normal BMI and no current smoking	426 (7.8)
No current smoking and non-sedentary behavior	273 (5.0)
Physical activity and no current smoking	273 (5.0)
Healthy diet, normal BMI and no current smoking	260 (4.8)
None	176 (3.2)
Physical activity, normal BMI, non-sedentary behavior and no current smoking	173 (3.1)
Healthy diet and no current smoking	149 (2.7)
Healthy diet, normal BMI, non-sedentary behavior and no current smoking	135 (2.4)
Physical activity, healthy diet, normal BMI and no current smoking	106 (1.9)
Normal BMI	94 (1.7)
Physical activity, non-sedentary behavior and no current smoking	93 (1.7)
Physical activity, healthy diet and no current smoking	62 (1.1)
All	61 (1.1)
Physical activity	61 (1.1)
Healthy diet, non-sedentary behavior and no current smoking	55 (1.0)
Physical activity, healthy diet, non-sedentary behavior and no current smoking	42 (0.7)
Non-sedentary behavior	33 (0.6)
Normal BMI and non-sedentary behavior	30 (0.5)
Physical activity and normal BMI	25 (0.4)
Healthy diet	22 (0.4)
Physical activity and non-sedentary behavior	15 (0.2)
Physical activity, normal BMI and non-sedentary behavior	12 (0.2)
Physical activity, healthy diet and normal BMI	8 (0.1)
Physical activity and healthy diet	8 (0.1)
Healthy diet and normal BMI	8 (0.1)
Physical activity, healthy diet and normal BMI	8 (0.1)
Physical activity, healthy diet and non-sedentary behavior	7 (0.1)
Healthy diet and non-sedentary behavior	5 (<0.1)
Physical activity, healthy diet, normal BMI and non-sedentary behavior	4 (<0.1)

Table S6. Association between single risk factors and risk of NAFLD.

Lifestyle Factor	OR (95% CI)	*P* value
Physical activity		
0	Ref	
1	0.84 (0.71, 1.00)	0.045
Leisure-time sedentary behavior		
0	Ref	
1	0.78 (0.66, 0.91)	0.002
BMI		
0	Ref	
1	0.12 (0.10, 0.14)	<0.001
Smoking		
0	Ref	
1	0.84 (0.68, 1.04)	0.106
Dietary quality		
0	Ref	
1	1.07 (0.88, 1.29)	0.493

Table S7. The lifestyle that included waist circumference rather than BMI and risk of NAFLD.

Healthy lifestyle score	Unadjusted model		Adjusted model[1]		Adjusted model[2]	
	OR (95% CI)	*P* value	OR (95% CI)	*P* value	OR (95% CI)	*P* value
0-1	Ref		Ref		Ref	
2	0.25 (0.19, 0.34)	<0.001	0.40 (0.30, 0.54)	<0.001	0.48 (0.35, 0.65)	<0.001
3	0.23 (0.16, 0.31)	<0.001	0.36 (0.26, 0.50)	<0.001	0.48 (0.34, 0.68)	<0.001
4-5	0.25 (0.16, 0.37)	<0.001	0.37 (0.24, 0.58)	<0.001	0.52 (0.32, 0.82)	0.005

NAFLD, non-alcoholic fatty liver disease; OR, odds ratio; CI, confidence interval; Ref, reference category; Estimated odds ratios (ORs) were compared with the scores are 0-1 group. [1] Adjusted for age, gender, education level, marriage status, and grades of employment. [2] Additionally adjusted for history of using statins, total cholesterol, triglyceride, fasting plasma glucose, total bilirubin, high-density lipoprotein cholesterol, and low-density lipoprotein cholesterol.

Table S8. Association of healthy lifestyle scores and risk of NAFLD among non-current drinkers.

Healthy Lifestyle Score	Unadjusted Model		Adjusted Model[1]		Adjusted Model[2]	
	OR (95% CI)	*P* value	OR (95% CI)	*P* value	OR (95% CI)	*P* value
0-1	Ref		Ref		Ref	
2	0.47 (0.36, 0.63)	<0.001	0.63 (0.47, 0.85)	0.002	0.72 (0.53, 0.99)	0.044
3	0.33 (0.24, 0.47)	<0.001	0.43 (0.30, 0.62)	<0.001	0.54 (0.38, 0.79)	0.001
4-5	0.29 (0.18, 0.47)	<0.001	0.37 (0.22, 0.60)	<0.001	0.51 (0.30, 0.84)	0.010

NAFLD, non-alcoholic fatty liver disease; OR, odds ratio; CI, confidence interval; Ref, reference category; Estimated odds ratios (ORs) were compared with the scores are 0-1 group. [1] Adjusted for age, gender, education level, marriage status, and grades of employment. [2] Additionally adjusted for history of using statins, total cholesterol, triglyceride, fasting plasma glucose, total bilirubin, high-density lipoprotein cholesterol, and low-density lipoprotein cholesterol.

Table S9. Contingency coefficient between individual lifestyle factors.

Contingency Coefficient	Smoking	BMI	Diet	Physical Activity	Sedentary Behavior
Smoking	1.00	0.151	0.035	0.013	0.031
BMI		1.00	0.029	0.004	0.039
Diet			1.00	0.067	0.079
Physical activity				1.00	0.048
Sedentary behavior					1.00
